# Quantifying the Core Deficit in Classical Schizophrenia

**DOI:** 10.1093/schizbullopen/sgaa031

**Published:** 2020-06-25

**Authors:** Mohanbabu Rathnaiah, Elizabeth B Liddle, Lauren Gascoyne, Jyothika Kumar, Mohammad Zia Ul Haq Katshu, Catherine Faruqi, Christina Kelly, Malkeet Gill, Sian Robson, Matt Brookes, Lena Palaniyappan, Peter Morris, Peter F Liddle

**Affiliations:** 1 Institute of Mental Health, University of Nottingham, Nottingham, UK; 2 Nottinghamshire Healthcare NHS Foundation Trust, Duncan McMillan House, Nottingham, UK; 3 Sir Peter Mansfield Imaging Centre, University of Nottingham, Nottingham, UK; 4 Department of Psychiatry and Robarts Research Institute, Western University, London, ON, Canada; 5 Lawson Imaging, Lawson Health Research Institute, London, ON, Canada

**Keywords:** schizophrenia, disorganization, negative symptoms, mental impoverishment, post-movement beta rebound, core deficit

## Abstract

In the classical descriptions of schizophrenia, Kraepelin and Bleuler recognized disorganization and impoverishment of mental activity as fundamental symptoms. Their classical descriptions also included a tendency to persisting disability. The psychopathological processes underlying persisting disability in schizophrenia remain poorly understood. The delineation of a core deficit underlying persisting disability would be of value in predicting outcome and enhancing treatment. We tested the hypothesis that mental disorganization and impoverishment are associated with persisting impairments of cognition and role function, and together reflect a latent core deficit that is discernible in cases diagnosed by modern criteria. We used Confirmatory Factor Analysis to determine whether measures of disorganization, mental impoverishment, impaired cognition, and role functioning in 40 patients with schizophrenia represent a single latent variable. Disorganization scores were computed from the variance shared between disorganization measures from 3 commonly used symptom scales. Mental impoverishment scores were computed similarly. A single factor model exhibited a good fit, supporting the hypothesis that these measures reflect a core deficit. Persisting brain disorders are associated with a reduction in post-movement beta rebound (PMBR), the characteristic increase in electrophysiological beta amplitude that follows a motor response. Patients had significantly reduced PMBR compared with healthy controls. PMBR was negatively correlated with core deficit score. While the symptoms constituting impoverished and disorganized mental activity are dissociable in schizophrenia, nonetheless, the variance that these 2 symptom domains share with impaired cognition and role function, appears to reflect a pathophysiological process that might be described as the core deficit of classical schizophrenia.

## Introduction

The classical concept of schizophrenia, developed by Kraepelin^1^ and Bleuler,^2^ has provided the basis for the classification of psychotic illnesses for more than a century, despite arguments that the concept of schizophrenia is outdated and perpetuates stigmatizing connotations of life-long disability.^3^ Nonetheless, many patients with psychotic illness do suffer persisting disabilities. Impairment of role function in “at-risk” and early-phase cases indicates that this is not merely a consequence of chronicity of illness or the effect of medication.^4^ Identifying the mechanisms underlying these disabilities would increase the likelihood of developing effective treatment.

The lack of a clear relationship between observed neurobiological abnormalities and clinical features based on current diagnostic criteria has prompted influential calls to abandon those diagnostic criteria in research studies, and instead seek biotypes based on neurobiological features.^5^ Before doing so, we should consider the possibility that current clinical criteria have not focused strongly enough on the relevant clinical features.

In the classical description, Kraepelin^1^ used the term *dementia praecox*, implying onset in young adult life and a tendency towards persisting symptoms and disabilities. He considered that weakened or disjointed volition were the core features of the disorder.^6^ Bleuler renamed *dementia praecox* “schizophrenia,” reflecting his perception of the disorder as a fragmentation of the mind.^2^ He considered that certain fundamental symptoms, most notably flattening of affect and loosening of associations, persist throughout the illness. Thus, both Kraepelin and Bleuler recognized persisting disorganization and impoverishment of mental activity as core symptoms of schizophrenia, and delusions and hallucinations as accessory features.

In recent decades, attempts to understand persisting disabilities focused on the positive-negative symptom dichotomy, embodied in the distinction between Type 1 and Type 2 schizophrenia.^7^ Type 1 schizophrenia was characterized by positive symptoms, including delusions and hallucinations, which respond to antipsychotic medications that block dopaminergic transmission. Type 2 schizophrenia was characterized by negative symptoms and cognitive impairment, which Crow proposed arose from structural damage to the brain, reflected in ventricular enlargement. However, the status of formal thought disorder was enigmatic within this framework, because its response to dopamine blockade is less clear-cut.^8^ As noted by Spohn et al,^9^ vague, wandering speech, reminiscent of Bleuler’s concept of loosening of associations, often persists despite treatment with antipsychotic medication.

The enigma of formal thought disorder was partially clarified by evidence from factor analysis that in the well-established phase of illness, the characteristic symptoms of schizophrenia segregate into 3 syndromes: reality distortion (delusions and hallucinations); disorganization (positive formal thought disorder, inappropriate affect, bizarre behavior); and core negative symptoms (flat affect, poverty of speech, decreased spontaneous movement).^10^ Because some authors regarded impaired role function and attentional impairment as negative symptoms, Liddle introduced the term “psychomotor poverty” to describe core negative symptoms reflecting impoverishment of mental activity. Furthermore, Liddle and colleagues demonstrated that psychomotor poverty and disorganization were separately associated with impaired role function and cognitive function.^10,11^

While the Bleulerian symptoms of psychomotor poverty and disorganization segregate into distinguishable dimensions in well-established schizophrenia, factor analyses of symptoms in early-phase cases of psychosis yield a single Bleulerian dimension embracing both impoverished and disorganized mental activity.^12,13^ This dimension is dissociated from reality distortion and also from affective symptoms. Moreover, in non-clinical samples and in cases at ultra-high risk of psychosis, symptoms of both disorganized and impoverished mental activity predict subsequent onset of psychotic symptoms, as well as persisting impairment of role function.^14,15^

A diverse range of cognitive impairments, particularly in the domains of attention, working memory, and speed of information processing, are well described in schizophrenia and are associated with poor outcome.^16^ There is also substantial evidence that cognitive impairment is related more strongly to disorganization and negative symptoms than to reality distortion.^17^

Thus, across the spectrum, from “at-risk” to established schizophrenia, impoverished and disorganized mental activity are consistently associated with long-term impairment of role function and cognitive impairment. In light of this evidence, Liddle^18^ proposed that psychomotor poverty and disorganization, together with associated cognitive and functional impairment, reflect an underlying pathological process that might reasonably be called the core deficit of classical schizophrenia.

However, despite its potential status as a feature of this core, disorganization is relatively difficult to quantify. There are no widely adopted procedures for assessing disorganization. Two commonly used symptom rating scales, the Positive and Negative Syndrome Scale (PANSS)^19^ and the Comprehensive Assessment of Symptoms and History (CASH),^20^ were developed in the era following Crow’s proposal regarding Type 1 and Type 2 schizophrenia^7^ and have a structure that focuses attention on positive and negative symptoms. Nonetheless, both scales include a diverse range of other symptoms. Liddle developed the Signs and Symptoms of Psychotic Illness (SSPI) scale, designed to embrace reality distortion, disorganization, and psychomotor poverty, in addition to features of depression and excitation,^21^ although this scale is less widely used.

These 3 different rating scales thus include different items representing impoverishment and disorganization of mental activity. It would be potentially of substantial value to establish whether consistent measures of disorganization and impoverishment can be derived from these symptom rating scales.

To investigate whether these 2 symptom dimensions reflect a core deficit that also impairs function and cognition, the assessment of cognition should capture a diverse range of functions. The MATRICS Consensus Cognitive Battery (MCCB) assesses a wide range of the cognitive dysfunctions that occur in schizophrenia.^22^ Digit symbol coding performance entails visual attention, active maintenance of symbol-digit pairings in working memory, and psychomotor speed and accounts for much of the variance in MCCB total score,^23^ consistent with an earlier meta-analysis reporting that performance on digit symbol coding tasks was the aspect of cognition most impaired in schizophrenia.^24^

Impoverished or disorganized mental activity has been associated with a diverse range of neurobiological abnormalities, at least in samples of medicated cases with chronic illness.^18^ These include abnormalities of the electrophysiological phenomenon of post-movement beta rebound (PMBR), a transient increase in the oscillatory power in the beta band occurring after the completion of a movement. Typically, PMBR follows a transient decrease in beta power known as Event-Related Beta Desynchronization (ERBD). The mechanisms of PMBR are not fully understood, but evidence suggests that it is involved in adaptive inhibition of neuronal networks following motor activity.^25^ It is modulated by bottom–up and top–down processes and is greater when the forward model that guides action is confirmed by sensory feedback.^26^

PMBR is reduced in schizophrenia, the magnitude of the reduction being correlated with overall severity of illness.^27^ Moreover, reduced PMBR was also associated with the disorganized and impoverished dimensions of schizotypy in a non-clinical sample,^28^ an association that can be neither attributed to confounding effects of medication, nor to the potentially damaging effects of chronicity of illness.

Motor abnormalities were common in schizophrenia prior to the antipsychotic era.^29^ Neuroimaging investigations have linked the emergence of these motor abnormalities to aberrant structure and function of cortical and subcortical components of the motor system.^30^ Furthermore, motor dysfunction predates the onset of the schizophrenia,^31,32^ and can be discernible in the first 2 years of life.^33^

PMBR is, therefore, a candidate neurobiological marker for the core deficit that we propose underlies persisting disability in classical schizophrenia.

We set out to delineate this putative core. The aims of this study were:

- To demonstrate that scores for 2 latent variables representing impoverishment and disorganization of mental activity in schizophrenia can be derived from each of 3 symptom rating scales, PANSS, SSPI, and CASH.
- To demonstrate that the variance shared between impoverishment, disorganization, cognitive impairment, and impaired role functioning reflects a single underlying latent variable reflecting a putative core deficit in schizophrenia.
- To demonstrate that this core deficit is associated with a neurobiological marker that reflects risk of persisting symptoms and disability.

## Methods

### Participants

This study is part of a multimodal imaging investigation of the relationship between clinical features of psychosis and brain structure/function. Patients aged 18–55 with a diagnosis of schizophrenia or schizoaffective disorder were referred to the study by community-based mental healthcare teams in Nottinghamshire, Derbyshire and Lincolnshire, England. Cases of schizoaffective disorder were included because of the proximity of schizoaffective disorder to schizophrenia on the schizophrenia spectrum, and the similarity in neuropsychological and neuroimaging correlates.^34^

Exclusion criteria were: (1) IQ below 70, (2) Lifetime history of substance dependence or harmful use in the past 6 months, (3) History of significant head trauma or medical conditions likely to have appreciable neurological or psychiatric effects, and (4) Contraindications for Magnetic Resonance Imaging (MRI) safety assessed by a standardized safety screening questionnaire. A personal or family history of psychotic illness was an exclusion criterion for controls.

Patients were included if: (1) they satisfied DSM IV criteria for schizophrenia or schizoaffective disorder^35^; this was determined by a consensus meeting in accordance with the best estimate procedure described by Leckman et al,^36^ utilizing evidence regarding current clinical state and a retrospective review of case notes and (2) they satisfied the criteria for stable phase of illness, defined as a change of no more than 10 points in their Social and Occupational Functioning Assessment Scale (SOFAS) score (defined in DSM-IV^35^) between assessment 6 weeks prior to and immediately prior to study participation

In light of the hypothesis that the putative core deficit is associated with persisting disabilities, we recruited cases during a stable phase of illness, defined as no change in SOFAS score of greater than 10 points nor change in psychotropic medication in the preceding 6 weeks. In the final sample of 40 patients, all but one were taking psychotropic medications. One patient had a diagnosis of schizoaffective disorder; the remainder had a diagnosis of schizophrenia.

In addition, 42 healthy controls, matching the patient sample group-wise by age and gender, were recruited by public advertisement, for the purpose of neurobiological comparison.

Written informed consent was obtained from all the subjects.

### Measures

Social and occupational functioning was assessed using SOFAS. IQ was assessed using the Quick Test,^37^ and cognition was assessed using a customized written and oral Digit Symbol Substitution Test (DSST),^38^ similar in format to the DSST from the Wechsler Adult Intelligence Scale.^39^

Defined daily dose (DDD) was computed for each patient’s antipsychotic medication dose. DDD is the assumed average maintenance dose per day for a drug used for its main indication in adults.^40^

A semi-standardized clinical interview designed to elicit the symptoms of psychotic illness required to score the SSPI^21^ was video-recorded. Symptoms were scored from the video-recordings by clinically trained raters, according to the scoring criteria for all 3 scales, respectively. For SSPI and PANSS, all symptom items were scored; for CASH, the aspects of appearance, behavior, and speech for symptom subscales relevant to the disorganization and impoverishment dimensions (table 1) were scored. We did not score items from the Avolition or Anhedonia CASH subscales as these are largely based on social and role function performance. The scorers (C.F., C.K., M.G., M.R.) together with PFL achieved good inter-rater reliability (α = .87 for PANSS total, α = .83 for SSPI total and α = .79 for CASH global items). Social and role function was scored according to the SOFAS (APA 1994) by a rater blind to symptom scores. Cognitive function was assessed using the customized DSST.

**Table 1.** Items Constituting Disorganization and Impoverishment Factors From 3 Scales

PANSS	SSPI	CASH
Disorganization		
P2 Conceptual disorganization	9. Attentional impairment	Bizarre behavior global
N5 Difficulty in abstract thinking	10. Disorientation	Positive formal thought disorder global
N7 Stereotyped thinking	14. Inappropriate affect	Catatonic motor behavior global
G5 Mannerism and posturing	17. Disordered form of thought	Inappropriate affect
G9 Unusual thought content	18. Peculiar behavior /mannerisms	Attention global
G10 Disorientation		
G11 Poor attention		
G13 Disturbance of volition		
G15 Preoccupation		
Impoverishment		
N1 Blunted affect	3. Anhedonia	Alogia global
N2 Emotional withdrawal	12. Underactivity	Affective flattening global
N3 Poor rapport	13. Flattened affect	
N4 Passive social withdrawal	16. Poverty of speech	
N6 Lack of spontaneity and flow		

*Note*: PANSS, Positive and Negative Syndrome Scale; SSPI, Signs and Symptoms of Psychotic Illness; CASH, Comprehensive Assessment of Symptoms and History.

### Quantifying Disorganization and Impoverishment

To generate specific measures of disorganization and impoverishment, we first performed a search of studies of symptom clustering that used the SSPI, PANSS, or CASH rating scales (see supplementary material, SA1 for details). From these, we selected a set of symptom measures that were assigned to either the disorganization or the impoverishment dimension, respectively, in at least 20% of the studies (table 1). We included the CASH attention subscale score in the disorganization dimension, as in the studies reviewed, it was more frequently associated with that dimension than with impoverishment. Then, for each rating scale, we computed total scores for each dimension by summing up the individual symptom measures. This yielded 2 variables (Disorganization and Impoverishment) for each rating scale. We also computed a Reality Distortion score for (the sum of SSPI delusion and hallucination scores).

### Post-Movement Beta Rebound

MEG data were acquired using a 275 channel whole head CTF system (MISL, Coquitlam, Canada) with a third-order synthetic gradiometer configuration, during a visuomotor task in which participants pressed a button with their right index finger at a self-paced regular rate during a 2-second presentation of a grating on the screen. Details of the MEG data acquisition and the visuomotor task have been reported previously.^27^ The MEG data were inspected for artifacts and pre-processed and further analysis was undertaken. From the group of 40 patients, 8 were excluded from the analysis of PMBR because no structural MRI brain scan was available, and 4 were excluded because of excessive movement. From the control group, 1 was excluded because no structural MRI scan was available, and one was excluded due to technical problems with the data acquisition. Thus the PMBR analysis was performed on 28 patients and 42 healthy controls.

The initial pre-processing of the MEG data included bandpass filtering of the data between 1 and 150 Hz, application of synthetic third-order gradiometers, and DC offset correction. Any trials containing large blinks or other artifacts were rejected by an investigator experienced in MEG analysis (L.G.). All data were epoched from 0 to 8.5 seconds relative to the onset of the visual grating. Head motion was calculated across the trial and any trials containing movement greater than 7 mm (Euclidean distance) from the starting point were rejected. Pre-processing was performed blind to group membership.

The pre-processed data were further analyzed using FieldTrip (version 20161011).^41^ The participant’s coregistered MRI was imported and segmented using FieldTrip’s default segmentation. The epoched MEG data were de-meaned per trial, bandpass filtered at 13–30 Hz using a 2-pass Butterworth filter, and downsampled to 300 Hz. Source localization was then performed using an LCMV beamformer on a 5 mm grid, warped to MNI template space using a singleshell forward model. The covariance matrix was constructed for the 13–30 Hz (beta) frequency band for a 0 to 8.5 seconds window post-grating onset.

We located the source of the beta signal associated with movement by finding the location, within left pre- or post-central gyrus, of greatest event-related beta desynchronization (ERBD) in the window 0.5 to 1.8 seconds after stimulus onset relative to a baseline period 7.0 to 8.3 seconds after stimulus onset. Pre- and post-central gyri were defined according to the AAL atlas.^42^ We then applied the previously calculated beamformer weights to extract the MEG timecourse data at that location.

This time course data was then high pass filtered (>1 Hz) at the full 600 Hz sampling rate. To quantify the post-movement beta rebound, we first selected a time window from 2.3 to 4.3 seconds after stimulus onset, which spanned the rebound peak in both the patient and control groups. We then computed the fast Fourier Transform (FFT) at a series of frequencies spanning the range 13–30 Hz in steps of 0.5 Hz within that time window using a Hanning filter and averaged the signal power across frequencies and trials for each participant. Similarly, we re-estimated the beta power in the baseline window and ERBD windows and expressed both PMBR and ERBD as percentage change from baseline value.

To quantify PMBR, we measured the percentage change of beta power occurring in a 2.3–4.3 seconds post-stimulus window relative to a baseline estimated in the 7–8.3 seconds post-stimulus interval within the 13–30 Hz frequency range for each participant. We also computed ERBD as the decrease in beta power in the window 0.5 to 1.8 seconds post-grating onset relative to the baseline period.

### Statistical Analyses

We derived composite scores for the Disorganization and Impoverishment symptom dimensions, respectively, using Principal Component Analysis (PCA). For each dimension, we entered the 3 scores into a PCA and derived factor scores for the first principle component, thus generating a composite score for that dimension reflecting variance shared by all 3 rating scales. Pearson correlation coefficients were computed between clinical scores.

IBM SPSS statistical software version 24.0 was used for all these statistical analyses. As the assumption of multivariate normality was violated for some pairwise correlations, and the assumptions of homogeneity of variance and normality of residuals violated for some comparisons between means, we computed bootstrapped (Bias Corrected accelerated; 5000 samples) estimates of confidence interval in order to determine statistical significance level.

We then used SPSS AMOS 24.0 to conduct a Confirmatory Factor Analysis (CFA) to test whether a single latent variable could account for the shared variance of the putative core deficit variables (composite Disorganization, composite Impoverishment, DSST, and SOFAS). Mardia’s multivariate skewness and kurtosis coefficients were calculated using WebPower (https://webpower.psychstat.org/). Factor extraction employed the maximum likelihood method. The threshold for modification indices was set at 4. Model fit was evaluated using indices of absolute fit, including the model chi-square test not being statistically significant, the goodness of fit index (GFI) ≥ .95 and the root mean square error of approximation (RMSEA) < .06.^43^

The regression imputation method was then used to derive factor scores for this model^44^ as a measure of the putative core deficit. To test whether the core deficit severity was associated with greater PMBR abnormality, we then computed the Pearson correlation between the core deficit score and PMBR. This computation was repeated with age and medication dosage and ERBD measures included as control variables.

Finally, to establish the practicality of estimating a measure of the core deficit from just one of the symptoms scale rather than a composite measure, we performed Maximum Likelihood factor analysis to compute core deficit scores using Disorganization and Impoverishment scores from one scale at a time, together with the DSST and SOFAS measures. We then compared these 3 core deficit scores (derived using each of the 3 rating scales) with the core deficit scores derived from the composite Disorganization and Impoverishment scores.

## Results

### Participants

Clinical and demographic features of the sample are presented in table 2. PMBR data was available for a subset of 28 patients, 21 of whom were included in the study by Robson et al,^27^ and for the 42 healthy controls. There were no statistically significant differences between this subset of patients and the healthy control groups in either age or gender representation.

**Table 2.** Clinical and Demographic Features of the Sample

	Patients All (*N* = 40)	Patients Subgroup^a^ (*N* = 28)	Controls (*N* = 42)	Patient Subgroup^a^ vs Controls
Gender (M/F)	30/10	21/7	28/14	χ ^2^ (1) = 0.556, ns
Age (y) *M* (*SD*)	28.08 (6.89)	27.14 (6.55)	27.89 (7.60)	*t* (68) = 0.469, ns
DSST Mean *M* (*SD*)	46.00 (10.93)	47.57 (11.69)	-	-
SOFAS *M* (*SD*)	58.80 (17.30)	57.94 (16.80)	-	-
Illness duration (mo) *M* (*SD*)	53.03 (45.64)	51.50 (45.40)	-	-
DDD *M* (*SD*)	1.17 (0.61)	1.23 (0.69)	-	-

*Note*: DDD, Defined daily dose; SOFAS, Social and Occupational Functioning Assessment Scale; DSST, Digit Symbol Substitution Test. One patient had a diagnosis of schizoaffective disorder, and was included in the PMBR subgroup. The remainder had a diagnosis of schizophrenia.

^a^Patient subgroup in whom PMBR measures were available.

### Correlations Between Clinical Scores in the Patient Group

All 3 Disorganization scores (PANSS, SSPI, CASH) were significantly correlated with each other, as were the 3 Impoverishment scores. Correlation coefficients and significance levels are given in table 3. The SSPI Reality Distortion score was not significantly correlated with any of the Disorganization or Impoverishment scores. Within each rating scale, Disorganization and Impoverishment scores were positively correlated. This correlation was statistically significant for PANSS, *r* = .61, *P* < .001, 95% CI (.407, .793) and SSPI, *r* = .37, *P* < .001, 95% CI (.173, .629) but did not reach significance for CASH, *r* = .30, ns, 95% CI (−.073, .602).

**Table 3.** Pearson Correlation Coefficients Between Each of the Measures of Disorganization (Upper Panel) and Impoverishment (Lower Panel): PANSS; SSPI; CASH; and the Composite Score Derived From PCAs of the 3 Measures

	PANSS	SSPI	CASH
	Disorganization		
SSPI	.859**		
CASH	.751**	.645**	
Composite	.955**	.916**	.868**
	Impoverishment		
SSPI	.903**		
CASH	.476*	.516**	
Composite	.932**	.945**	.725**

*Note*: PANSS, Positive and Negative Syndrome Scale; SSPI, Signs and Symptoms of Psychotic Illness; CASH, Comprehensive Assessment of Symptoms and History; PCA, Principal Component Analysis.

**Bootstrapped *P* value for correlation <.001.

In the PCA of the 3 Disorganization scores, the first PC had an eigenvalue of 2.51 and accounted for 83.6% of the variance. Both other components had eigenvalues of less than 1. All 3 rating scale scores loaded strongly on the first PC. Factor scores for this PC were, therefore, defined as a composite Disorganization score. In the PCA of the 3 Impoverishment scores, the first PC had a similarly high eigenvalue (2.29) and accounted for 76.2% of the variance. Again, both other components had eigenvalues of less than 1, and all 3 rating scale scores loaded strongly on the first PC. The factor scores for this PC was therefore defined as a composite Impoverishment score. Correlations between these composite scores and the individual rating scale scores are also shown in table 3.

The composite scores for Disorganization and Impoverishment were strongly correlated with each other, *r* = .526, *P* < .001, 95% CI (.326, .710). Neither were significantly correlated with SSPI Reality Distortion score. Both composite scores were significantly correlated with DSST scores (Disorganization: *r* = −.332, *P* < .05, 95% CI [−.613, −.068]; Impoverishment: *r* = −.312, *P* < .05, 95% CI [−.598, −.039]). Disorganization was significantly correlated with SOFAS score, *r* = .480, *P* < .01, 95% CI (−.684, −.228). The Impoverishment score correlation with SOFAS scores did not reach significance, *r* = −.296, *P* < .1, 95% CI (−.544, −.008). DSST and SOFAS scores were not significantly correlated with each other.

### Confirmatory Factor Analysis

Our single factor model consisted of measures of the 4 putative features of core deficit, namely, composite Disorganization, composite Impoverishment, DSST, and SOFAS. Multivariate normality tests indicated a violation of the assumption of multivariate normality (Mardia’s coefficient for skewness = 5.88, *P* = .006; kurtosis = 24.21, *P* = .93). As DSST scores were positively skewed within the patient group, we substituted the natural log of DSST score. This restored multivariate normality (Mardia’s coefficient values both nonsignificant: skewness = 4.06, *P* = .234; kurtosis = 22.11, *P* = .389).

**Fig. 1.**
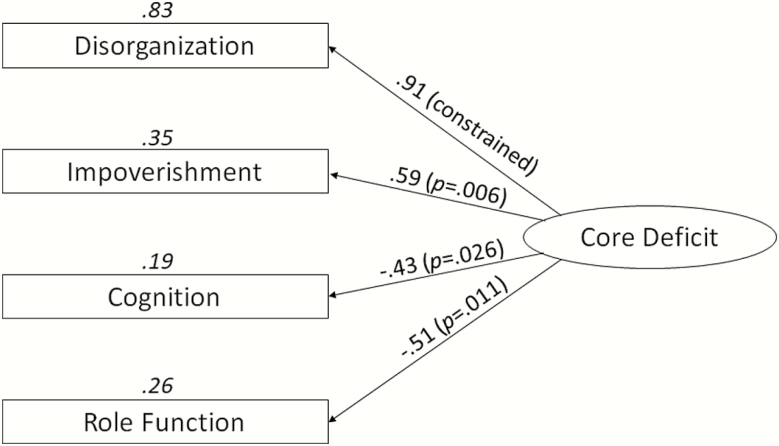
Estimated regression weights from confirmatory factor analysis for the putative core deficit. Variables were: Disorganization (Composite Disorganization measure); Impoverishment: (composite Impoverishment measure); Cognition (log of DSST scores) and Role Function (SOFAS scores). Values next to the arrows are the standardized regression weights with significance value. Values in italics above the variable boxes are the squared multiple correlations (*R*^2^). SOFAS, Social and Occupational Functioning Assessment Scale; DSST, Digit Symbol Substitution Test.

Fit indices for the single factor model indicated a good fit: χ ^2^ (2) = 1.817, *P* = .403; RMSEA < .001 GFI = .979. The modification indices were <4, indicating that modifications to the model were unlikely to improve the fit. The single factor accounted for 52.6% of the total variance, and 40.6% of the shared variance. Regression weights were scaled to the Disorganization scores, and scaled regression weights for all the other variables were significantly different from zero (figure 1).

**Fig. 2.**
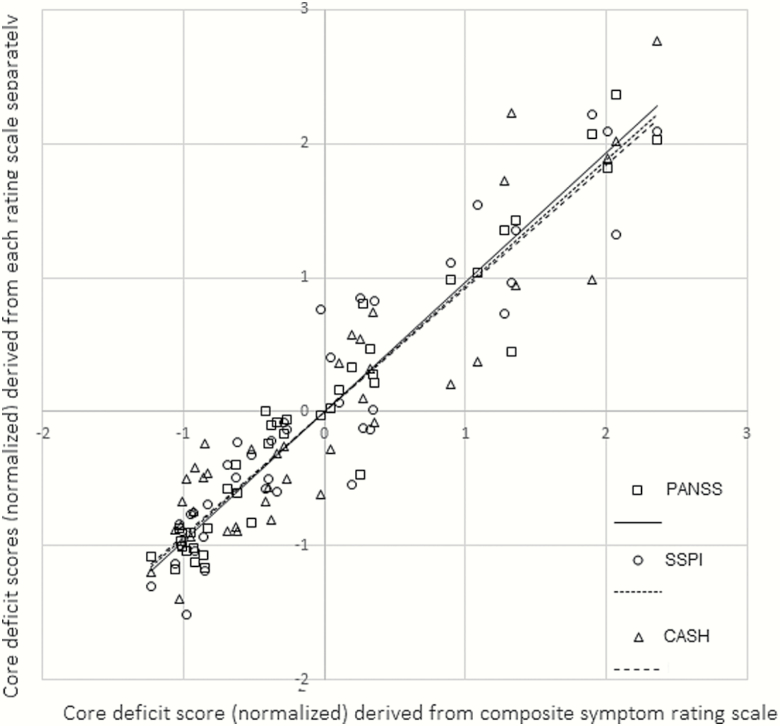
Normalized core deficit Factor scores from Confirmatory Factor Analysis (CFA) using composite symptom scores for Disorganization and Impoverishment (horizontal axes) plotted against normalized factor scores derived from factor analyses using PANSS, SSPI, and CASH rating scales, respectively. In all factor analyses, Role Function scores (SOFAS) and Cognition scores (log of DSST scores) were included in the model. PANSS, Positive and Negative Syndrome Scale; SSPI, Signs and Symptoms of Psychotic Illness; CASH, Comprehensive Assessment of Symptoms and History; SOFAS, Social and Occupational Functioning Assessment Scale; DSST, Digit Symbol Substitution Test.

When factor analyses were performed using the symptom scores from each rating scale separately, instead of the composite scores, the factor scores were strongly correlated with the scores obtained using composite scores (PANSS: *r* = .964, *P* < .001, 95% CI [.927, .987]; SSPI: *r* = .938, *P* < .001, 95% CI [.891, .967]; CASH: *r* = .919, *P* < .001, 95% CI [.865, .954]). A scatterplot is shown in figure 2, and details of the additional factor analyses are presented in the supplementary material (SA2 and ST1–ST3).

### Post-Movement Beta Rebound

PMBR was significantly reduced in the schizophrenia group compared to healthy controls, *t* (68) = 3.55, *P* < .001, 95% CI (19.5, 69.3).

**Fig. 3.**
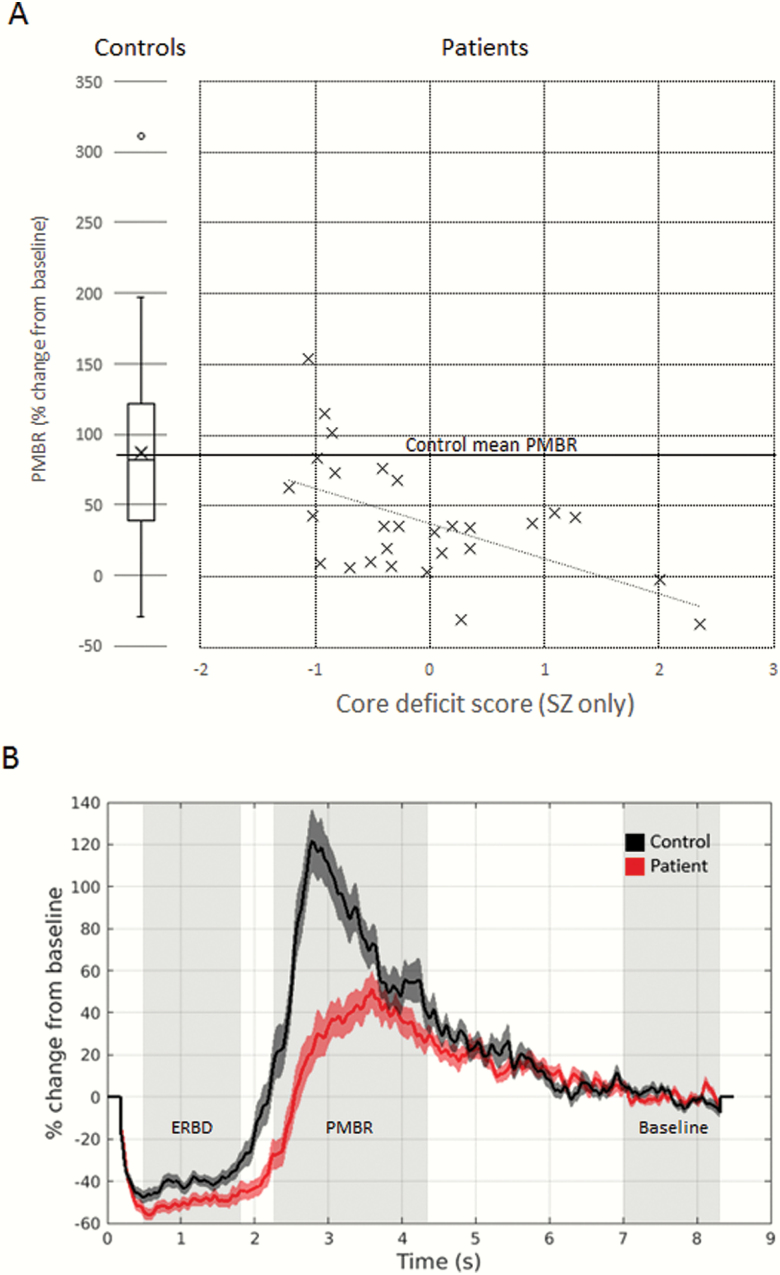
(A) PMBR scores (vertical axis) plotted against Core Deficit scores in the patient group. A box plot showing the distribution of PMBR scores in healthy control participants is shown on the left for comparison. (B) evolution of beta power averaged across trials and participants within each group. Shaded areas represent Event-Related Beta Desynchronization (ERBD), PMBR, and Baseline time windows, respectively. Time 0 is the onset of the stimulus.

Within the patient group, PMBR was significantly and negatively correlated with the CFA factor scores representing the Core Deficit score, *r* = −.543, *P* < .01, 95% CI (−.730, −.261) indicating that high core deficit scores were associated with reduced PMBR. Figure 3A plots Core Deficit scores against PMBR, and shows the distribution of the PMBR values for healthy controls for comparison. Figure 3B shows the average time evolution of the beta signal for each group.

The correlation between Core Deficit scores and PMBR remained significant after controlling for age, medication (DDD) and ERBD, *r*(23) = −.513, *P* < .01, 95% CI (−.718, −.274). PMBR was not significantly correlated with the SSPI Reality Distortion scores, *r* = .222, ns.

With regard to the 4 variables included in the CFA, PMBR was significantly correlated with the composite Disorganization score, *r* = −.521, *P* < .001, 95% CI (−.711, −.243) with Role Function (SOFAS), *r* = .569, *P* < .01, 95% CI (.262, .785) and with Cognition (log DSST), *r* = .349, *P* < .05, 95% CI (.040, .604) but not with composite Impoverishment, *r* = −.355, ns.

In light of the possibility that the relationship between the 4 variables that constitute the putative core deficit have been inflated by the inclusion of clinical assessments of cognitive function in the Disorganization scores for, we computed adjusted Disorganization scores, omitting the PANSS scores for attention (G11) and difficulty in abstract thinking (N5), CASH attention impairment and SSPI attentional impairment from estimates of disorganization for the 3 rating scales. We then computed an adjusted composite Disorganization score that does not include a direct contribution from the clinical scores for attention and abstract thinking. The relevant correlates of the adjusted composite Disorganization score were very similar to that for the original unadjusted composite Disorganization score. The correlation between log DSST and adjusted composite Disorganization; *r* = .321 (*P* < .05; *df* = 39). The CFA yielded similar loadings when the adjusted composite Disorganization score was entered. The correlation between the resulting adjusted Core Deficit score and PMBR was −.577 (*P* = .001, *df* = 27).

## Discussion

Our findings demonstrate that scores for the 2 latent variables representing impoverishment and disorganization of mental activity in schizophrenia can be derived from each of 3 symptom rating scales, PANSS, SSPI, and CASH. Despite differences between scales in the content of the items contributing to these scores, the 3 scales yield strongly correlated scores for each of these symptom clusters. However, the strength of correlation between Psychomotor Poverty and Disorganization scores was appreciably greater for PANSS than for the other 2 scales. It is likely that this reflects the fact that several individual symptom items within PANSS are defined in a manner that embraces impoverishment and disorganization of mental activity within a single item. For example, blunted affect and inappropriate affect are scored within a single item in PANSS, whereas they are scored separately in CASH and SSPI. It is likely that there are both shared and distinct aspects of the pathophysiological processes generating inappropriate affect or flattened affect.

The shared variance between measures of mental impoverishment, disorganization, cognitive impairment, and impaired role function can be accounted for by a single latent variable that can reasonably be described as a core deficit of classical schizophrenia. Furthermore, similar estimates of this core deficit can be derived using any of the 3 symptom rating scales.

Impoverishment and disorganization have been recognized as fundamental features of schizophrenia since the time of Kraepelin and Bleuler. In current practice, delusions and hallucinations are regarded a cardinal features. The relationship of the core deficit with delusions and hallucinations requires clarification. In our sample, the core deficit scores were not correlated significantly with severity of concurrent reality distortion. This observation is consistent with the results of factor analysis of symptoms in well-established illness^10^ and in the early phase of illness.^12,13^ However, case note review indicated that all of the cases in our sample had experienced delusions and/or hallucination at some time in the course of their illness. Furthermore, in their prospective study of a non-clinical sample of young people, Dominguez et al^14^ found that mental impoverishment and disorganization predicted risk of subsequent overt psychosis and subsequent poor functional outcome. Ziermans et al^15^ found that disorganization in individuals at high risk of psychosis predicted severity of subsequent persisting disabilities. Overall, the evidence indicates that impoverishment and disorganization predispose to an illness characterized by episodes of acute psychosis and a tendency towards persisting disabilities.

The observation that impoverishment and/or disorganization are associated with a predisposition to the reality distortion typical of overt psychosis raises the question of the relationship between “classical” schizophrenia and the modern concept of schizophrenia reflected in the Diagnostic and Statistical Manual,^35^ which places greater emphasis on reality distortion. It is plausible that cases with the “classical” core deficit form a subset of cases defined by modern criteria. Alternately the classical core deficit might best be regarded as a dimension of schizophrenia. A larger sample would be required to distinguish between these possibilities.

The specificity of cognitive impairment in schizophrenia remains a topic of debate.^45^ Cognitive impairment occurs in many neuropsychiatric conditions. Nonetheless, when cognitive impairment occurs in conjunction with mental impoverishment and/or disorganization in schizophrenia, it adds valuable information about the likelihood of persisting disability. The fact that DSST provides an overall estimate of multiple aspects of cognitive function does not allow us to conclude that any specific aspect of cognition is preferentially associated with the putative core deficit.

The demonstration that the putative core deficit is correlated with the reduction in PMBR provides evidence that the core deficit is associated with identifiable brain dysfunction. In particular, it indicates a relationship with an abnormality associated with disturbed long-range connectivity in the brain.^46^ It should be noted that reduced PMBR has been reported in other neuropsychiatric conditions, including autism^47^ and fronto-temporal dementia,^48^ which indicates that the relationship is not specific to schizophrenia. However, our finding in this study, together with the evidence that the magnitude of PMBR is inversely correlated with severity of mental disorganization and impoverishment in a non-clinical sample^28^ raises the possibility that PMBR might nonetheless be a sensitive measure of risk of persisting symptoms and disability in both the early and later phases of a psychotic illness.

Robson et al^27^ examined PMBR in an overlapping sample of cases. However, whereas Robson et al, examined the correlation of PMBR with overall illness severity without distinguishing between core deficit features and reality distortion, this study has demonstrated that the core deficit, but not reality distortion, is associated with PMBR.

While the possibility that the inclusion of clinical measures of cognitive function in the Disorganization scores might in principle have contributed to the observed relationship between Disorganization and DSST, the computation of Disorganization scores after omitting the scores for the items reflecting the clinical assessment of cognitive function led to a very similar value for the correlation between Disorganization and log DSST, and to similar loadings on the factor representing the putative Core Deficit and a similar relationship between Core Deficit and PMBR. Furthermore, it should be noted that items directly assessing occupational and social function had been omitted for the CASH scores. Thus the evidence indicates that the relationship between the scores for the phenomena constituting the Core Deficit are not largely accounted for by an overlap of the measures.

The limitations of our study include a sample size that was inadequate to allow testing of more complex models in the CFA. PMBR data was not available in all cases. Although current dose of medication did not account for the relationship between the core deficit and PMBR, we cannot exclude possible effects of sustained exposure to antipsychotic medication.

Recent studies of cognitive remediation therapy in schizophrenia have reported benefits extending beyond cognition to improvements in negative symptoms and social functioning.^49^ This suggests that cognitive remediation might be effective in alleviating the core deficit, although meta-analyses^50,51^ suggest that effect sizes may be modest. Insofar as diminished PMBR might reflect impaired long-range connectivity in specific brain circuits, it is plausible that therapeutic efficacy might be enhanced by combining cognitive remediation with neuromodulatory techniques such as transcranial direct current stimulation (tDCS) or transcranial magnetic stimulation (TMS) targeted on the relevant circuits. Further investigation of the neural mechanism of the putative core deficit is a priority. If the concept is validated, the construction of an assessment instrument with a balance of items tapping each contributory domain would be both clinically useful and elicit item-level data that would help further refine the factor structure.

## Supplementary Material

sgaa031_suppl_Supplementary_Material

## References

[1] Kraepelin E. Dementia Praecox and Paraphrenia. Barclay RM, Trans. Edinburgh, UK: ES Livington; 1919.

[2] Bleuler E. Dementia Praecox or the Group of Schizophrenias. Zinken H, Trans. New York, NY: International Universities Press; 1950.

[3] Guloksuz S, van Os J The slow death of the concept of schizophrenia and the painful birth of the psychosis spectrum. Psychol Med. 2018;48(2):229–244. 28689498 10.1017/S0033291717001775

[4] Lee SJ, Kim KR, Lee SY, An SK Impaired social and role function in ultra-high risk for psychosis and first-episode schizophrenia: its relations with negative symptoms. Psychiatry Investig. 2017;14(5):539–545. 10.4306/pi.2017.14.5.539 PMC5639120 29042877

[5] Insel TR The NIMH Research Domain Criteria (RDoC) project: precision medicine for psychiatry. Am J Psychiatry. 2014;171(4):395–397. 24687194 10.1176/appi.ajp.2014.14020138

[6] Kraepelin E Patterns of mental disorder. transl. In: Hirsch SR, Shepherd M, Marshall H, eds. Themes and Variations in European Psychiatry. Bristol, UK: Wright; 1974:7–30.

[7] Crow TJ Molecular pathology of schizophrenia: more than one disease process? Br Med J. 1980;280(6207):66–68. 6101544 10.1136/bmj.280.6207.66 PMC1600263

[8] Johnstone EC, Crow TJ, Frith CD, Carney MW, Price JS Mechanism of the antipsychotic effect in the treatment of acute schizophrenia. Lancet. 1978;1(8069):848–851. 76797 10.1016/s0140-6736(78)90193-9

[9] Spohn HE, Coyne L, Larson J, Mittleman F, Spray J, Hayes K Episodic and residual thought pathology in chronic schizophrenics: effect of neuroleptics. Schizophr Bull. 1986;12(3):394–407. 2876514 10.1093/schbul/12.3.394

[10] Liddle PF The symptoms of chronic schizophrenia. A re-examination of the positive-negative dichotomy. Br J Psychiatry. 1987;151:145–151. 3690102 10.1192/bjp.151.2.145

[11] Liddle PF, Morris DL Schizophrenic syndromes and frontal lobe performance. Br J Psychiatry. 1991;158:340–345. 2036532 10.1192/bjp.158.3.340

[12] McGorry PD, Bell RC, Dudgeon PL, Jackson HJ The dimensional structure of first episode psychosis: an exploratory factor analysis. Psychol Med. 1998;28(4):935–947. 9723148 10.1017/s0033291798006771

[13] Tonna M, Ossola P, Marchesi C, et al.; GET UP Group Dimensional structure of first episode psychosis. Early Interv Psychiatry. 2019;13(6):1431–1438. 30644165 10.1111/eip.12789

[14] Dominguez MD, Saka MC, can Saka M, Lieb R, Wittchen HU, van Os J Early expression of negative/disorganized symptoms predicting psychotic experiences and subsequent clinical psychosis: a 10-year study. Am J Psychiatry. 2010;167(9):1075–1082. 20634371 10.1176/appi.ajp.2010.09060883

[15] Ziermans T, de Wit S, Schothorst P, et al. Neurocognitive and clinical predictors of long-term outcome in adolescents at ultra-high risk for psychosis: a 6-year follow-up. PLoS One. 2014;9(4):e93994. 24705808 10.1371/journal.pone.0093994 PMC3976376

[16] Green MF, Horan WP, Lee J Nonsocial and social cognition in schizophrenia: current evidence and future directions. World Psychiatry. 2019;18(2):146–161. 31059632 10.1002/wps.20624 PMC6502429

[17] Ventura J, Wood RC, Hellemann GS Symptom domains and neurocognitive functioning can help differentiate social cognitive processes in schizophrenia: a meta-analysis. Schizophr Bull. 2013;39(1):102–111. 21765165 10.1093/schbul/sbr067 PMC3523911

[18] Liddle PF The core deficit of classical schizophrenia: implications for predicting the functional outcome of psychotic illness and developing effective treatments. Can J Psychiatry. 2019;64(10):680–685. 31434513 10.1177/0706743719870515 PMC6783668

[19] Kay SR, Fiszbein A, Opler LA The positive and negative syndrome scale (PANSS) for schizophrenia. Schizophr Bull. 1987;13(2):261–276. 3616518 10.1093/schbul/13.2.261

[20] Andreasen NC, Flaum M, Arndt S The Comprehensive Assessment of Symptoms and History (CASH). An instrument for assessing diagnosis and psychopathology. Arch Gen Psychiatry. 1992;49(8):615–623. 1637251 10.1001/archpsyc.1992.01820080023004

[21] Liddle PF, Ngan ET, Duffield G, Kho K, Warren AJ Signs and Symptoms of Psychotic Illness (SSPI): a rating scale. Br J Psychiatry. 2002;180:45–50. 11772851 10.1192/bjp.180.1.45

[22] Nuechterlein KH, Green MF, Kern RS, et al. The MATRICS consensus cognitive battery, part 1: test selection, reliability, and validity. Am J Psychiatry. 2008;165(2):203–213. 18172019 10.1176/appi.ajp.2007.07010042

[23] Burton CZ, Vella L, Harvey PD, Patterson TL, Heaton RK, Twamley EW Factor structure of the MATRICS Consensus Cognitive Battery (MCCB) in schizophrenia. Schizophr Res. 2013;146(1–3):244–248. 23507359 10.1016/j.schres.2013.02.026 PMC3740948

[24] Dickinson D, Ramsey ME, Gold JM Overlooking the obvious: a meta-analytic comparison of digit symbol coding tasks and other cognitive measures in schizophrenia. Arch Gen Psychiatry. 2007;64(5):532–542. 17485605 10.1001/archpsyc.64.5.532

[25] Solis-Escalante T, Müller-Putz GR, Pfurtscheller G, Neuper C Cue-induced beta rebound during withholding of overt and covert foot movement. Clin Neurophysiol. 2012;123(6):1182–1190. 22349305 10.1016/j.clinph.2012.01.013

[26] Cao L, Hu YM Beta rebound in visuomotor adaptation: still the status quo? J Neurosci. 2016;36(24):6365–6367. 27307225 10.1523/JNEUROSCI.1007-16.2016 PMC6601919

[27] Robson SE, Brookes MJ, Hall EL, et al. Abnormal visuomotor processing in schizophrenia. Neuroimage Clin. 2016;12:869–878. 27872809 10.1016/j.nicl.2015.08.005 PMC5107643

[28] Hunt BAE, Liddle EB, Gascoyne LE, et al. Attenuated post-movement beta rebound associated with schizotypal features in healthy people. Schizophr Bull. 2019;45(4):883–891. 30239878 10.1093/schbul/sby117 PMC6581139

[29] Rogers D The motor disorders of severe psychiatric illness: a conflict of paradigms. Br J Psychiatry. 1985;147:221–232. 2866007 10.1192/bjp.147.3.221

[30] Walther S, Strik W Motor symptoms and schizophrenia. Neuropsychobiology. 2012;66(2):77–92. 22814247 10.1159/000339456

[31] Kindler J, Schultze-Lutter F, Michel C, et al. Abnormal involuntary movements are linked to psychosis-risk in children and adolescents: results of a population-based study. Schizophr Res. 2016;174(1–3):58–64. 27160790 10.1016/j.schres.2016.04.032

[32] Mittal VA, Walker EF Movement abnormalities predict conversion to Axis I psychosis among prodromal adolescents. J Abnorm Psychol. 2007;116(4):796–803. 18020725 10.1037/0021-843X.116.4.796

[33] Walker EF, Savoie T, Davis D Neuromotor precursors of schizophrenia. Schizophr Bull. 1994;20(3):441–451. 7526446 10.1093/schbul/20.3.441

[34] Madre M, Canales-Rodríguez EJ, Ortiz-Gil J, et al. Neuropsychological and neuroimaging underpinnings of schizoaffective disorder: a systematic review. Acta Psychiatr Scand. 2016;134(1):16–30. 27028168 10.1111/acps.12564

[35] American Psychiatric Association. Diagnostic and Statistical Manual of Mental Disorders (4th Ed.). 4th ed. Washington, DC: American Psychiatric Publishing, Inc.; 1994.

[36] Leckman JF, Sholomskas D, Thompson WD, Belanger A, Weissman MM Best estimate of lifetime psychiatric diagnosis: a methodological study. Arch Gen Psychiatry. 1982;39(8):879–883. 7103676 10.1001/archpsyc.1982.04290080001001

[37] Ammons RB, Ammons CH The Quick Test (QT): provisional manual. Psychol Rep. 1962;11(1):111–161.

[38] Palaniyappan L, Al-Radaideh A, Mougin O, Gowland P, Liddle PF Combined white matter imaging suggests myelination defects in visual processing regions in schizophrenia. Neuropsychopharmacology. 2013;38(9):1808–1815. 23558741 10.1038/npp.2013.80 PMC3712891

[39] David WDW. “Wechsler Adult Intelligence Scale–Fourth Edition (WAIS–IV). 2008th ed. San Antonio, TX: Psychological Corporation; 2008.

[40] WHO Collaborating Centre for Drug Statistics Methodology. Guidelines for ATC Classification and DDD Assignment 2013. Oslo, Norway: WHO; 2012.

[41] Oostenveld R, Fries P, Maris E, Schoffelen JM FieldTrip: open source software for advanced analysis of MEG, EEG, and invasive electrophysiological data. Comput Intell Neurosci. 2011;2011:156869. 21253357 10.1155/2011/156869 PMC3021840

[42] Tzourio-Mazoyer N, Landeau B, Papathanassiou D, et al. Automated anatomical labeling of activations in SPM using a macroscopic anatomical parcellation of the MNI MRI single-subject brain. NeuroImage. 2002;15(1):273–289. 11771995 10.1006/nimg.2001.0978

[43] Schreiber JB, Stage FK, King J, Nora A, Barlow EA Reporting structural equation modeling and confirmatory factor analysis results: a review. J Educ Res. 2006;99(6):323–337.

[44] DiStefano C, Zhu M, Mindrila D Understanding and using factor scores: considerations for the applied researcher. Pract Assess Res Eval. 2009;14(20):1–11.

[45] Barch DM Nonsocial and social cognitive function in psychosis: interrelationships, specificity and innovative approaches. World Psychiatry. 2019;18(2):117–118. 31059631 10.1002/wps.20653 PMC6502400

[46] Barratt EL, Tewarie PK, Clarke MA, et al. Abnormal task driven neural oscillations in multiple sclerosis: a visuomotor MEG study. Hum Brain Mapp. 2017;38(5):2441–2453. 28240392 10.1002/hbm.23531 PMC6866959

[47] Honaga E, Ishii R, Kurimoto R, et al. Post-movement beta rebound abnormality as indicator of mirror neuron system dysfunction in autistic spectrum disorder: an MEG study. Neurosci Lett. 2010;478(3):141–145. 20452402 10.1016/j.neulet.2010.05.004

[48] Hughes LE, Rittman T, Robbins TW, Rowe JB Reply: brain oscillations, inhibition and social inappropriateness in frontotemporal degeneration. Brain. 2018;141(10):e74. 30212860 10.1093/brain/awy235

[49] Ventura J, Subotnik KL, Gretchen-Doorly D, et al. Cognitive remediation can improve negative symptoms and social functioning in first-episode schizophrenia: a randomized controlled trial. Schizophr Res. 2019;203:24–31. 29128326 10.1016/j.schres.2017.10.005 PMC6589092

[50] Cella M, Preti A, Edwards C, Dow T, Wykes T Cognitive remediation for negative symptoms of schizophrenia: a network meta-analysis. Clin Psychol Rev. 2017;52:43–51. 27930934 10.1016/j.cpr.2016.11.009

[51] Wykes T, Huddy V, Cellard C, McGurk SR, Czobor P A meta-analysis of cognitive remediation for schizophrenia: methodology and effect sizes. Am J Psychiatry. 2011;168(5):472–485. 21406461 10.1176/appi.ajp.2010.10060855

