# Supplementary material for: Quantifying the Core Deficit in Classical Schizophrenia
Source: Schizophr Bull Open. 2020 Jun 25;1(1):sgaa031. doi: 10.1093/schizbullopen/sgaa031 (PMC7418866; doi:10.1093/schizbullopen/sgaa031)
Supplement: sgaa031_suppl_Supplementary_Material [file sgaa031_suppl_supplementary_material.docx]

**Supplementary Material**

Contents

[Appendices 1](#_Toc16806221)

[SA1: Systematic literature review for disorganization and impoverishment item 1](#_Toc16806223)

[SA2: Supplementary results 3](#_Toc16806225)

[Tables 3](#_Toc16806226)

[ST1: Factor loadings for Core Deficit items based on symptoms assessed using PANSS 3](#_Toc16806227)

[ST2: Factor loadings for Core Deficit items based on symptoms assessed using SSPI 4](#_Toc16806228)

[ST3: Factor loadings for Core Deficit items based on symptoms assessed using CASH 4](#_Toc16806229)

[References 4](#_Toc16806230)

# Appendices

## SA1: Systematic literature review for disorganization and impoverishment items

We searched Embase (via Ovid), MEDLINE (via PubMed), and PsycINFO (via Ovid) on 5 July 2019. We employed a search strategy designed to identify studies that used factor analyses to investigate the relationships between symptoms assessed using the three rating scales of interest. The search terms were refined by reviewing relevant reviews, ⁶′⁷ and examining controlled vocabularies from databases (MeSH, Emtree, and APA Thesaurus). An information specialist (FS) designed and tested the search strategies against the existing collection of papers from previous reviews. We ran the following search for MEDLINE in PubMed and we adopted it for the other databases:

((Schizophrenia/Diagnosis[MH] OR Schizophrenia/Complications[MH] OR "Schizophrenia, Childhood/Diagnosis"[MH] OR (Schizophrenic Psychology[MH] AND Psychiatric Status Rating Scales[MH]) OR ("Psychiatric Status Rating Scales/Statistics and Numerical Data"[MH] AND Psychometrics[MH])) AND ("Factor Analysis, Statistical"[MH] OR Component Structure*[TI] OR Factor Model*[TI] OR Factor Structure*[TI] OR Factorial Structure*[TI] OR Symptom Dimension*[TI] OR Symptom Structure*[TI])) OR ((Psychotic Disorders/Diagnosis[MH] OR Schizophrenia/Diagnosis[MH] OR (Schizophrenia[MH] AND Syndrome[MH])) AND ("Factor Analysis, Statistical"[MH] OR "Models, Psychological"[MH] OR ((Cluster Analysis[MH] OR "Models, Psychological"[MH] OR "Models, Statistical"[MH] OR Principal Component Analysis[MH]) AND Psychiatric Status Rating Scales[MH]) OR Factor Analysis[TI] OR Formal Though*[TIAB] OR Principal Component Analys*[TIAB])) OR ((Psychotic Disorders/Diagnosis[MH] OR Schizophrenia, Disorganized/Diagnosis[MH] OR Schizophrenia[MH] OR Schizophrenia[TI]) AND ((Signs[TIAB] AND Symptoms[TIAB] AND Psychotic[TIAB] AND Illness[TIAB]) OR SSPI[TIAB] OR Psychomotor Poverty[TIAB]))

We augmented the search by examining the reference sections of selected papers. Details of all the articles selected will be published elsewhere. Briefly, all the studies which reported the factor loadings from factor analysis of the items in each of the three scales were selected. Comprehensive Assessment of Symptoms and History (CASH) includes items from the Scale for the assessment of positive symptoms (SAPS) and the Scale for the Assessment of Negative Symptoms (SANS). Studies reporting factor loadings derived from CASH or from SANS and SAPS (more than 20 in number) were selected. Numerous studies reporting factor loadings for the Positive and Negative Symptom Scale (PANSS) were found (more than 80 in number). In the case of Signs and Symptoms of Psychotic Illness (SSPI) scale, there were fewer studies reporting factors analyses. Therefore studies which reported scoring disorganization and impoverishment using a specified combination of relevant items were included (more than 10 in number),

From the selected studies for each of the scales, the items reported in at least 20% of the published studies were included in deriving the composite measure for disorganization and impoverishment for each scale.

## SA2: Supplementary results

We performed three separate factor analyses of the four core deficit items (disorganization, impoverishment, log DSST and SOFAS) using disorganization and impoverishment scores derived from each of the three different symptom rating scales (PANSS, SSPI and CASH) in turn. Factors were extracted using the Maximum Likelihood procedure, and factors with eigenvalues greater than unity were retained.

In all three analyses, a single factor was retained. In each case the eigenvalue of the first factor was greater than 2.4 while the eigenvalues of all other factors were less than 0.6.

The proportion of total variance accounted for by the first factor was 71.9% for PANSS; 70.2% for SSPI; and 61.6% for CASH. The proportion of shared variance accounted for by the first factor was 62.9% for PANSS; 60.5% for SSPI; and 48.9% for CASH.

# Tables

## ST1: Factor loadings for Core Deficit items based on symptoms assessed using PANSS

|  | Factor 1 |
| --- | --- |
| PANSS disorganization | .891 |
| PANSS impoverishment | .795 |
| Log DSST | -.710 |
| SOFAS | -.765 |

##

## ST2: Factor loadings for Core Deficit items based on symptoms assessed using SSPI

|  | Factor 1 |
| --- | --- |
| SSPI disorganization | .858 |
| SSPI impoverishment | .714 |
| Log DSST | -.718 |
| SOFAS | -.811 |

## ST3: Factor loadings for Core Deficit items based on symptoms assessed using CASH

|  | Factor 1 |
| --- | --- |
| CASH disorganization | .680 |
| CASH impoverishment | .663 |
| Log DSST | -.688 |
| SOFAS | -.762 |

#

# References

1. Peralta, V., Cuesta, M.J., 2001. How many and which are the psychopathological dimensions in schizophrenia? Issues influencing their ascertainment. *Schizophr Res.* 49(3), 269-285.
2. Shafer, A., Dazzi, F., 2019. Meta-analysis of the positive and Negative Syndrome Scale (PANSS) factor structure. *J Psychiatric Res*. 115, 113-120.
